# Drug Delivery Systems and Flavonoids: Current Knowledge in Melanoma Treatment and Future Perspectives

**DOI:** 10.3390/mi13111838

**Published:** 2022-10-27

**Authors:** Catarina Cunha, Ana L. Daniel-da-Silva, Helena Oliveira

**Affiliations:** 1 Department of Biology, CESAM—Centre for Environmental and Marine Studies, University of Aveiro, 3810-193 Aveiro, Portugal; 2 Department of Chemistry, CICECO—Aveiro Institute of Materials, University of Aveiro, 3810-193 Aveiro, Portugal

**Keywords:** nanocarriers, drug delivery systems, flavonoids, melanoma

## Abstract

Melanoma is an aggressive form of skin cancer with a high prevalence in the population. An early diagnosis is crucial to cure this disease. Still, when this is not possible, combining potent pharmacological agents and effective drug delivery systems is essential to achieve optimal treatment and improve patients’ quality of life. Nanotechnology application in biomedical sciences to encapsulate anticancer drugs, including flavonoids, in order to enhance therapeutic efficacy has attracted particular interest. Flavonoids have shown effectiveness against various types of cancers including in melanoma, but they show low aqueous solubility, low stability and very poor oral bioavailability. The utilization of novel drug delivery systems could increase flavonoid bioavailability, thereby potentiating its antitumor effects in melanoma. This review summarizes the potential of different flavonoids in melanoma treatment and the several nanosystems used to improve their biological activity, considering published information that reported improved biological and pharmacological properties of encapsulated flavonoids.

## 1. Introduction

Melanoma is a malignant tumor that arises from melanocytes and is one of the most aggressive and deadliest forms of skin cancer [1,2]. In the early stages, the principal therapeutic option for melanoma is surgery, but in advanced phases, melanoma becomes very resistant to the existing therapies, leading to a severe prognosis [1,3]. Conventional treatments for melanoma involve tumor excision, combined with chemotherapy, immunotherapy, and radiotherapy [1,4]. Conventional melanoma therapy often fails due to poor accessibility to tumor tissues, insufficient specificity, and toxic side effects [1]. Additionally, tumors may also develop drug resistance; therefore, new therapeutic approaches are essential to improve cancer treatment and enhance patients’ quality of life.

In this context, flavonoids, such as curcumin, quercetin and apigenin, among others, gained significant interest due to their broad spectrum of properties, which include anti-inflammatory, antioxidant and antiviral activity [5,6]. Still, the most important is perhaps their anticarcinogenic activity, affecting the regulation of cell proliferation and cell-cycle progression, induction of apoptosis and inhibition of tumor angiogenesis [7,8].

Flavonoids are phenolic phytochemicals that have shown effectiveness against various types of cancers in different in vitro assays and animal models [9,10], including in melanoma cells [6,11,12]. Despite in vitro and in vivo promising results, there are some concerns in using flavonoids in biological environments, which include low aqueous solubility, low stability and very poor oral bioavailability [13]. Recent advances in the field of nanotechnology offer an opportunity to encapsulate antitumor drugs in nanocarrier systems. This approach can help to overcome some limitations of the applicability of flavonoids, increasing their bioavailability, solubility, stability and allowing a controlled and prolonged release, ensuring a targeted action and consequently reducing side effects and improving their effectiveness [14,15,16]. A wide range of nanoparticles are available to encapsulate drugs and are classified based on their constituents. Liposomes, ethosomes, solid lipid nanoparticles, micelles, polymeric nanoparticles and metal-based nanoparticles are examples of drug delivery systems capable of carrying and delivering flavonoids [14]. The use of nanoparticles may enhance flavonoid properties. In addition, the shape, size and surface characteristics of the nanomaterials can be optimized to increase the targeting specificity of nanoparticles to the tumor microenvironment [17,18]. Various studies showed the successful use of nanoparticles in cancer treatment using different biological models. Liu et al. developed a magnetic nanosystem for the delivery of apigenin that showed good biocompatibility and stability after modification with mesoporous silica (SiO_2_) and hyaluronic acid (HA) as targeting ligand. Additionally, cell viability assay also showed that the nanosystem induced a higher cell inhibition effect than the free apigenin [19]. The effect was ascribed to the surface amination of SiO_2_, which increased the apigenin loading capacity, and to the delay of the release caused by HA introduction. Similarly, the encapsulation of curcumin [20] and quercetin [21] in mesoporous SiO_2_ nanoparticles resulted in biocompatible materials with an enhanced antiproliferation effect on cancer cells compared to the nonencapsulated flavonoid.

Herein we present an overview of the applicability of flavonoids in melanoma treatment. First, we introduce the melanoma subtypes and the existing treatments, followed by a brief description of different flavonoids and nanocarriers and their applications in melanoma therapy. The main objective of this review is to resume intuitively the current knowledge of several flavonoids encapsulated in nanoparticles in melanoma treatment and present–future research perspectives.

## 2. Melanoma Skin Cancer

Skin cancers are more prevalent among Caucasians and are classified into three major types: cutaneous malignant melanoma, basal cell carcinoma (BCC) and squamous cell carcinoma (SCC). BCC and SCC skin cancers are classified as nonmelanocytic skin cancers and form a subcategory known as nonmelanocytic skin cancers (NMSC) [3,22].

Melanoma (melanocytic skin cancer) constitutes one of the most aggressive forms of skin cancer [3]. It has its origin in genetically altered melanocytes, cells located at the base of the epidermis specialized in melanin synthesis that contributes to skin color and protection against various exterior aggressions such as UV radiation, free radicals and potentially toxic chemicals [23,24,25]. Melanoma can be classified into four main types of clinicopathologic cellular subtypes: (a) superficial spreading melanoma, which is the most common subtype found on the surface of the skin; (b) nodular melanoma, which appears like spots and grows quickly; (c) lentigo maligna melanoma, which appears less commonly and may arise due to extended sun exposure; and (d) acral lentiginous melanoma, which is the rarest type and may not be related with sun exposure [3].

Some melanomas arise due to genetic propensity, such as mutations in *BRAF*, *RAS* and *NF1* genes [3,26], but others, as mentioned above, are related to sun exposure. UVB radiation can cause direct DNA damage that may contribute to inducing mutations in keratinocytes, upregulation of gene expression and suppression of immune reactions, leading to the development of skin cancer in humans [26,27]. Most melanoma mutations are part of the mitogen-activated protein kinase (MAPK) and phosphatidylinositol-3 kinase pathway (PI3K) [28,29].

Regarding melanoma treatment, surgery is still the method of choice in the early stages. When melanoma is diagnosed in advanced phases, chemotherapy, immunotherapy, radiotherapy and targeted therapy are the other treatment options [1,30]. Common cytotoxic drugs for melanoma treatment include the alkylating agent dacarbazine and its derivative temozolomide, carboplatin and antimitotic drug paclitaxel, among others [22,31,32]. Due to the invasiveness and metastasis ability of malignant melanoma and despite the results shown by these conventional therapies, some limitations diminish the efficacy of the treatments [1,22,28]. Tumor resistance to drugs, toxic side effects such as nausea and fatigue, limited specificity and poor accessibility to tumor tissues complicate the treatment with these conventional therapies [30,33]. Considering melanoma’s heterogeneity, complexity and aggressive behavior is crucial to maximize therapeutics with minimal toxic side effects.

## 3. Flavonoids

Flavonoids are a large group of polyphenolic compounds found in a wide range of vegetables, fruits and medicinal herbs [24,34], subdivided into isoflavonoids, flavanones, flavanols, flavonols, flavones and anthocyanidins [8,35]. These compounds have attracted much attention due to their antioxidant, anti-inflammatory, antiproliferative and chemoprotective efficacies, especially important in cancer treatment [5,22,24]. Flavonoids exhibit an antimelanoma effect by inhibiting cell proliferation and invasion and inducing apoptosis [24]. The mechanisms are also multi-effect, through free radicals scavenging, cellular metabolism and cell cycle regulation, epigenetic modification including DNA methylation and histone deacetylation [24,35].

**Figure 1:**
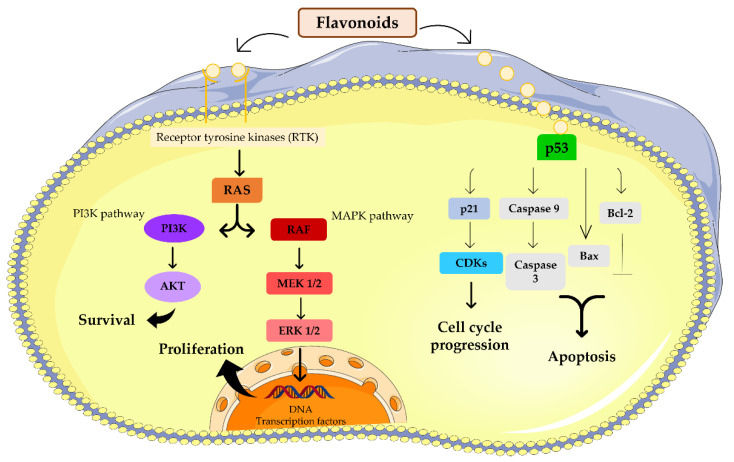
Molecular targets of flavonoids. Flavonoids target PI3K and the MAPK signaling pathway, inducing survival and proliferation of tumor cells. Additionally, flavonoids can also affect p53 and influence cell cycle progression by regulation of cyclin dependent kinases (CDKs) and by apoptosis.

In general, flavonoids interact with several molecular targets involved in melanoma pathogenesis (Figure 1), such as p53, Bcl-2 [9,36], the MAPK pathway, caspase 3 and 9 [37], mitogen-activated protein kinase (MEK)/extracellular signal-regulated kinase (ERK) [38], the phosphatidylinositol 3-kinase (PI3K)/Akt pathway [39,40] and the cyclin-dependent kinase pathway (Cdk) [22,34,41]. Recently, a study reported that flavonoids blocked the two-pore channel 2 (TPC2) present in the melanosome membrane, which increased melanin production and reduced proliferation, migration and invasion of melanoma cells [42]. All these findings support the use of flavonoids as novel compounds in melanoma treatment.

**Figure 2:**
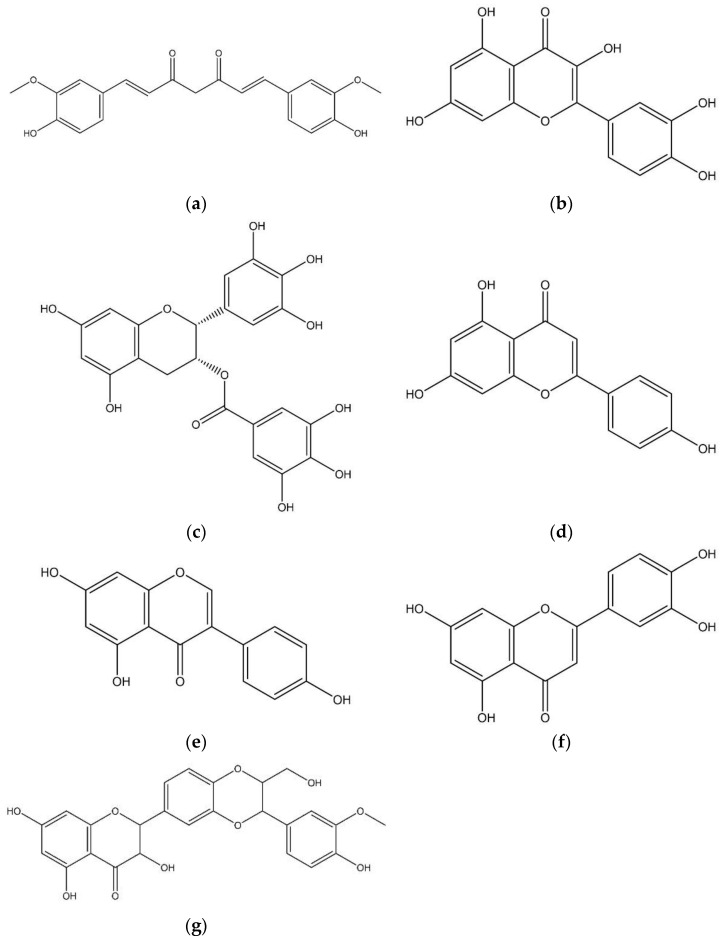
Flavonoid chemical structure. (**a**) Curcumin; (**b**) quercetin; (**c**) epigallocatechin-3-gallate (EGCG); (**d**) apigenin; (**e**) genistein; (**f**) luteolin; (**g**) silymarin.

Here we summarize important information regarding curcumin, quercetin, epigallocatechin-3-gallate (EGCG), apigenin, genistein, luteolin and silymarin that must be taken in account when flavonoids are used in cancer treatment, and below we present the flavonoid chemical structure (Figure 2).

Curcumin (Figure 2a) is a polyphenol extracted from the plant *Curcuma longa* that appears to have an important role in several biological processes and pharmacological properties that could be advantageous in treating human diseases [6,43]. Like other flavonoids, curcumin has very poor aqueous solubility, limiting its use with maximal benefits [43]. The chemopreventive efficacy of curcumin has been demonstrated in vitro in different types of cancer, including breast, lung, colon, brain and skin, both in its free or encapsulated forms [20,44,45,46,47,48]. Additionally, it was reported that curcumin could reverse multidrug resistance mechanisms by its capacity of regulating signaling pathways in cancer cells and reducing the expression of proteins related to drug resistance [49,50,51]. The aforementioned studies suggest that the incorporation of curcumin into nanoformulations increased its biological properties, which may indicate that curcumin could be used as a novel therapeutic compound in melanoma treatment [43].

Quercetin (Figure 2b) is one of the abundant dietary flavonoids in green and black tea, fruit and vegetables and is partially soluble in water [5,52]. Quercetin appears to have selectivity toward melanoma tumor cells [53]. It was reported that free quercetin inhibited melanoma cell migration and invasion in vitro [54]. Furthermore, data suggest that quercetin encapsulation improves its bioavailability and stability [21,55,56]. Therefore, this flavonoid is a valuable compound that could be used in melanoma treatment.

Epigallocatechin-3-gallate (EGCG) (Figure 2c) is the major component in green tea leaves and has anticarcinogenic, antiproliferative, antioxidant and antiangiogenic activity [57,58]. Several studies using melanoma cell lines showed that EGCG can induce apoptosis, reduce cell proliferation and inhibit cell invasion and migration [59,60,61]. Despite its biological properties, EGCG is unstable; therefore, its incorporation in a drug delivery system will enhance its stability in biological systems and improve its anticancer effects [62,63].

Apigenin (Figure 2d) is mainly found in fruits and vegetables and can be isolated from the buds and flowers of *Hypericum perforatum* [52,64]. Apigenin can induce the apoptotic process in melanoma cells by activation of mitochondrial apoptotic pathway caused by alterations in mitochondrial membrane and increased activity of caspases [12,65]. Furthermore, one study found apigenin caused DNA fragmentation indicating an early apoptotic stage [65]. In another study, it was shown that apigenin decreased the invasion of cells in vitro and inhibited melanoma growth and metastatic potential in vivo [54]. Although it has low water solubility that results in poor cell uptake, apigenin encapsulation increases its antiproliferative potential, solubility, dissolution and bioavailability, making this a promising flavonoid for melanoma therapy [19,65,66,67].

Genistein (Figure 2e) is an isoflavonoid mainly found in soybean [52,68]. Genistein showed anticancer effects such as a decrease in the tumor size, reduction in metastatic potential and induction of apoptosis [69,70]. Still, the clinical application of genistein is restricted due to its poor bioavailability and low solubility. The use of nanocarriers makes it possible to take advantage of genistein in anticancer treatment, as demonstrated in several studies using different cell models (colon and colorectal cancer, adenocarcinoma) [52,71,72,73,74].

Luteolin (Figure 2f) belongs to the flavonoids group flavones and is found in fruits and vegetables. It has a wide range of pharmacological actions, specifically anti-inflammatory activity, that could be related to its anticancer properties [34,75]. The anticancer properties include, for example, induction of apoptosis or inhibition of cell proliferation, which involves DNA damage, production of ROS and angiogenesis, among others [34,68]. Luteolin also has antioxidant and antimicrobial activity, as shown by Kazmi and colleagues [5]. Additionally, promising results were shown in various in vitro studies using different cell lines (colorectal, skin and brain) where solubility was improved using nanosystems [5,76,77,78]. Still, more investigation is needed, in particular for melanoma.

Silymarin (Figure 2g) is another flavonoid used as anticancer agent. This flavonoid is extracted from the seeds and fruits of *Sylibum marianum* [79]. Silymarin has antioxidant properties [80] and anticancer capacity, namely by inducing cell cycle arrest and melanoma cell growth inhibition [38,81,82]. As mentioned, flavonoids’ poor aqueous solubility results in poor absorption and bioavailability; therefore, silymarin loading into nanostructures can make it more bioavailable and may enhance its anticancer effects, for example, by the activation of apoptosis [83,84].

## 4. Nano-Based Delivery Systems of Flavonoids for Melanoma Treatment

**Figure 3:**
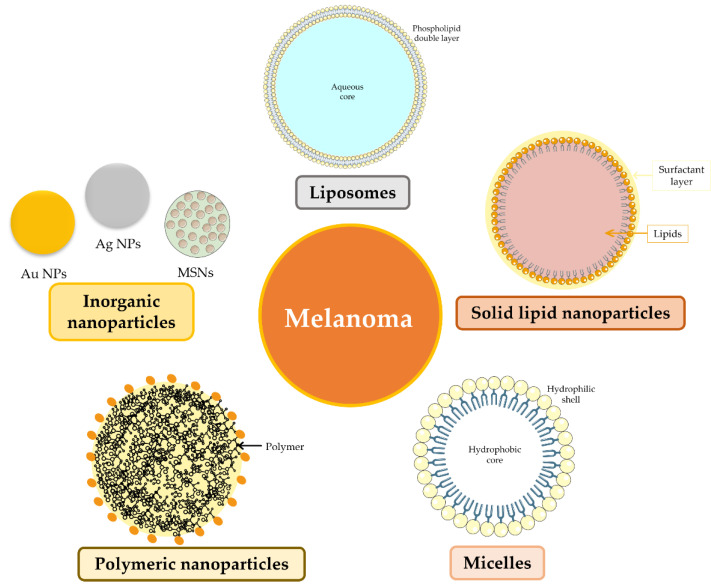
Drug delivery systems used in melanoma therapy.

The use of nanotechnology in medicine is rapidly increasing as it may help to circumvent to the limitations of conventional therapies, namely toxic side effects, multiple drug resistance and nonspecific targeting. Nanotechnology is particularly useful in overcoming some challenges presented by free drugs, such as poor water solubility and rapid degradation in blood circulation [1,3]. The development of different nanoengineered systems loaded with single or multiple anticancer drugs, which can be further functionalized with small molecules, peptides or antibodies increasing the target specificity toward cancer cells, opens new perspectives for potential treatment for melanoma [3,30,71,85]. Several drug delivery systems have been designed to enhance flavonoid bioavailability, stability and their therapeutic efficacy in melanoma therapy [3], such as liposomes [86,87], solid lipid nanoparticles [6], micelles [88], polymeric nanoparticles [65] and inorganic nanoparticles such as mesoporous silica or metal-based [89] (Figure 3). Table 1 lists various studies that combined these drug delivery systems with flavonoids for melanoma therapy.

Liposomes are lipid vesicles composed of a phospholipid double layer with an intern aqueous compartment where hydrophilic drugs can be loaded [14,99]. Due to these characteristics, liposomes are usually used for topical drug delivery on skin [87,92]. Despite this, liposomes can be inoculated to carry flavonoids such as quercetin [100] and luteolin [101] and be used in cancer treatment. Liposomes have been shown to be very effective in melanoma treatment due to biodegradability, low toxicity, hydrophobic and hydrophilic characteristics and small size [86,87,92,99]. Additionally, in vivo studies showed that liposomes reduce the cytotoxicity induced by curcumin and quercetin and reduced the inflammation/oxidative stress associated with precancerous/cancerous skin lesions [102] (see Table 1).

Solid lipid nanoparticles (SLNs) are composed of lipids that are solid at room temperature, a surfactant layer surrounding the solid lipid layer and, in the interior, active pharmaceutical ingredients [103]. SLNs have numerous properties such as controlled drug release, increased drug stability and bioavailability, reproducibility and large-scale production, in addition to their high payload capacity and nontoxic nature [6,94,103,104]. Despite these advantages, this nanocarrier is not much studied carrying flavonoids, but it was reported carrying curcumin [6,94]. These studies showed that the anticancer properties of curcumin were enhanced using SLNs and modified SLNs, which may indicate that these nanocarriers could be used with other flavonoids.

Micelles have two distinct regions, a hydrophobic core and a hydrophilic shell, essential properties to encapsulate hydrophobic drugs in the core or hydrophilic drugs in the shell [22,76]. These nanocarriers are also biodegradable and nontoxic, making them very attractive to encapsulate drugs [105]. Curcumin was loaded in micelles modified with mPEG-PLA (methoxy-poly(ethylene glycol)-poly(D,L-lactide)), and this nanosystem was found to have improved curcumin aqueous solubility and bioavailability, which consequently induced higher cytotoxicity in melanoma cells when compared to free curcumin [88,93]. In addition, curcumin mPEG-PLA micelles induced more apoptosis in melanoma cells than the free curcumin and inhibited neovascularization in tumor tissues (see Table 1) [88]. Micelles encapsulating luteolin and fisetin increased the solubility in water of these flavonoids, showing that this nanoformulation could be used for clinical applications [76,106].

Polymeric nanoparticles are prepared using polymers and could have a spherical or irregular shape [104]. These NPs have high drug-loading capacity, high biocompatibility and good drug release control [17,104], useful characteristics to encapsulate flavonoids. Polylactic acid (PLA) and polylactic acid-glycolic acid copolymer (PLGA) are commonly used in drug delivery systems to improve water solubility and poor stability of polyphenol [65,104]. Additionally, polymers can be combined with each other. Recently, biopolymers such as chitosan have received significant attention due to their properties such as low toxicity, biocompatibility and biodegradability [107]. In addition, chitosan abundance, natural availability and flexibility make it very attractive to be used in biomedical sciences as a drug delivery system for polyphenols [72,107]. When conjugated with folic acid, it induces changes in melanoma cells [108]. Under low pH conditions, chitosan dissolves easily, which increases its use in cancer research due to an acidic tumor microenvironment [109,110]. Apigenin and curcumin encapsulated in polymeric nanoparticles revealed that polyphenols’ antimetastatic and antiproliferative effects were improved due to enhanced bioavailability allowed by the nanoformulation [65,66,91].

Mesoporous silica nanoparticles (MSNs) have gained wide popularity over recent years due to their high surface area and pore volume, easy surface functionalization, biocompatibility and degradability in biological environments and also high level of clearance and excretion [84,110,111]. MSN characteristics make them ideal nanocarriers for encapsulation and delivery of flavonoids. Several studies showed the therapeutic efficacy of different drugs loaded in MSNs such as quercetin [56] and genistein [73] in colorectal and in colon cancer cells, respectively. These nanoparticles were functionalized with folic acid due to high expression of folate receptor in tumor cells [56] and with PEG [73], increasing the affinity of nanoparticles to cancer cells and potentiate the flavonoids’ anticancer properties. Bioavailability of silymarin was enhanced in an in vivo study, which also showed oral administration of MSNs as a promising alternative [84]. There have been other studies using different cell models that showed the versatility and potential of MSNs encapsulating resveratrol [112], curcumin [44,45,46,48], EGCG [57,113] and apigenin [19,114]. Bollu et al. synthesized and characterized with success silica-based mesoporous materials with curcumin that exhibited higher toxicity against the B16-F10 melanoma cell line than the pristine curcumin [20]. Another study using curcumin showed similar results, namely that MSNs enhanced solubility, sustained release profile, and improved cell cytotoxicity toward a skin cancer cell line [46] (see Table 1). Sapino and colleagues used quercetin-loaded MSNs for topical delivery and concluded that the nanoformulation was more efficient than quercetin alone, causing about 50% inhibition of cell proliferation [21]. In melanoma cells, it was shown that MSNs as nanocarriers have impact on different aspects of cellular function including cell proliferation, apoptosis, cytoskeleton formation, adhesion and migration confirming the potential of MSNs as efficient drug delivery nanocarriers and therapeutic systems [115].

Finally, metal-based nanoparticles have recently attracted attention in the cancer treatment field. These nanoparticles are mainly based in noble metals, especially gold (Au), silver (Ag) and platinum (Pt), and can be used as therapeutic agents, as contrast agents for diagnosis or as nanocarriers [116,117]. Gold nanoparticles (Au NPs) exhibit excellent biocompatibility and chemical stability and versatility in size, shape and surface that allows an easy functionalization [117]. Au NPs’ size and shape can be tailored to favor the absorption of long-wavelength light (usually near-infrared light) and convert it into heat, being extremely helpful in biomedical sciences specially for photothermal or photodynamic applications [17,104,117]. These nanoparticles have the capacity to remodel the tumor microenvironment, making it more prone to therapy. For example, after heat or light irradiation, reactive oxygen species are produced, which sensitizes the tissue and can reduce tumor cell viability, including in melanoma cells [17,118,119]. A study used liposome gold nanoparticles for the delivery of curcumin (Au-Lipos Cur NPs) that acted as an in situ adjuvant for photothermal treatment of melanoma [95]. Singh et al. demonstrated that using Au-Lipos NPs increased the cytotoxicity on the B16-F10 melanoma cells after laser irradiation (Table 1). In another study, authors investigated polyethylene glycol-curcumin-gold nanoparticles (PEG-Cur-Au NPs) for photothermal therapy and concluded that after laser irradiation these NPs were capable of drastically reducing B16-F10 melanoma tumors both in vitro and in vivo [96]. Silver nanoparticles (Ag NPs) are among the most widely used nanomaterials in medical field, despite some concerns about their toxicity toward normal cells [117,120,121]. Having this in consideration, the surface functionalization to stabilize the nanoparticles as reported by Netchareonsirisuk and colleagues [122] is crucial. In this study, Ag NPs capped with alginate were selectively toxic to the A375 human malignant melanoma cell line but not to the normal cell line. The authors suggested that the toxicity of Ag NPs depended on the capping agent and the type of cell line [122], but more studies are needed to better understand Ag NPs to be used in melanoma treatment.

Overall, the nanoencapsulation of flavonoids enhances their bioavailability, stability and solubility. However, the number of studies devoted to flavonoid delivery for melanoma therapy is still scarce. Polymeric and lipid-based nanoparticles are the most studied, which may be due to the low intrinsic solubility of inorganic materials [123]. Among polymeric nanoparticles, chitosan was a common choice [48,97,123]. Owing to amine groups, chitosan is pH-sensitive and can facilitate drug release at the acidic environment of a tumor cell. Nevertheless, other polymers could be of interest for skin cancer treatment [124,125] but remain unexplored for flavonoid delivery. Liposomes also showed good results by increasing the inhibitory effect of curcumin on melanoma cells [92]. This could be related to liposomes’ principal component, phospholipids, that have good biocompatibility and could promote the drug delivery through the cell membrane, increasing the drug concentration in the cells [92]. Similar results were obtained using micelles [88,93], showing good biocompatibility and improved cytotoxicity in melanoma cells. Solid lipid nanoparticles also improved bioavailability of flavonoids by crossing the blood–brain barrier (BBB) after oral administration [94]. Among inorganic nanocarriers, mesoporous silica nanoparticles are of great interest because of their large surface area and porosity that allows the entrapment of drug molecules for potential later release. The pore size can be adjusted during MSN synthesis (2 to 50 nm) to increase loading capacity and tailor the release profile. Several flavonoids are photosensitive and prone to oxidation, and the encapsulation in MSNs improved their chemical stability [46]. Importantly, the entrapment of flavonoids onto MSN pores can lead to flavonoid amorphization, increasing aqueous solubility and bioavailability [112]. Silica-based nanocarriers, including MSNs, found several therapeutic applications for skin diseases [126], being regarded as biocompatible [46,90]. The in vitro studies confirmed that these nanoparticles are safe [21,46,90], but before its clinical use, in vivo tests must be performed. Less investigated for the delivery of flavonoids are gold-based nanoparticles, which are exciting systems with the ability to convert long-wavelength light into heat, which in combination with flavonoids might have an anticancer multi-effect.

Flavonoids can be co-delivered with other compounds. In Barui and colleagues’ study [86], due to cancer cell resistance to ceramides, curcumin was used in combination with ceramides to reverse the drug resistance shown by cancer cells, and a synergistic therapeutic benefit from simultaneous delivery of curcumin and a homoserine-based ceramide was demonstrated [86]. Another example of co-delivery is the work of Jose et al. [87]. In this case, curcumin was co-delivered with anti-STAT3 (signal transducer and activator of transcription 3) siRNA using liposomes. The combination of curcumin and STAT3 siRNA resulted in significantly greater cancer cell growth inhibition compared with curcumin or STAT3 siRNA alone [87]. These results are similar to Tavakoli et al. [91], who demonstrated the simultaneous use of curcumin and chrysin, and to Palliyage et al. [6], who used curcumin and resveratrol. Both studies showed that the drug combination induced higher anticancer effects on melanoma cells than the drugs alone. It should be noted that curcumin is one of the most studied flavonoids, both used alone and in co-delivery.

## 5. Conclusions

Melanoma skin cancer is highly metastatic, and its rate increasing worldwide makes the development of new therapeutic approaches is essential. Phytochemicals, particularly flavonoids, showed to be effective against different cancer cell lines, including melanoma. Flavonoids target multiple signaling pathways critical to the pathogenesis of melanoma, being curcumin, quercetin, epigallocatechin-3-gallate, apigenin, genistein, luteolin and silymarin potential adjuvants, to treat metastatic melanoma. Nanoencapsulation of flavonoids allows us to surpass some limitations of the free flavonoids by enhancing their bioavailability, stability and cell-targeting specificity, which increases the efficacy at low concentrations compared to the free drugs, contributing to a reduction of inadvertent side effects. Curcumin has been loaded in different types of nanocarriers and is comparatively the most extensively studied flavonoid in melanoma therapy. Polymeric nanoparticles, liposomes and inorganic nanoparticles are the most investigated nanoparticles, and despite the modifications on the nanoparticles surface that make them more compatible and with affinity toward cancer cells, more studies are needed to explore the interaction between these NPs and healthy cells and tissues. Still, in general, all the flavonoids highlighted in this review have their antitumor effect enhanced when in a nanoformulation. Therefore, despite the research emphasized in this review, it is clear that more studies are needed both in vitro and in vivo in order to assess the effects of flavonoids and combinations of flavonoids loaded into different drug delivery systems in order to determine which combinations yield the best results.

## Figures and Tables

**Table 1:** Summary of various flavonoids in the management of melanoma loaded in different nanosystems.

Nanosystem	Biological Model	Key Studies	Concluding Remark	Ref.
**Curcumin**				
Mesoporous silica nanoparticles (MSNs)	SCC-25 cells	Encapsulate curcumin in the nanopores of MSNs and compare the cytotoxic effects of free curcumin	Curcumin loaded inside MSNs showed enhanced solubility, sustained release profile and improved cell cytotoxicity compared to the pure drug.	[46]
MSNs	B16-F10 cells	Synthesize and characterize two mesoporous silica materials (MSU-2 and MCM-41) for the delivery of curcumin	The silica-based mesoporous materials are biocompatible. Curcumin loaded in silica-based materials showed more inhibition of cell proliferation than free curcumin.	[20]
Polymeric nanoparticles	A375 cells	Investigate the antitumor properties of silica-encapsulated curcumin nanoparticles (SCNP) and chitosan with silica co-encapsulated curcumin nanoparticles (CSCNP)	CSCNP showed higher cytotoxicity in treated tumor cells. Nanoencapsulation of curcumin with silica and chitosan increased curcumin stability and enhanced its cytotoxic activity.	[48]
Polymeric nanoparticles (chitosan)	B16-F10 cells C57BL6 mouse model	Prepare chitosan-coated polycaprolactone (PCL) nanoparticles containing curcumin and evaluate the antimetastatic activity both in vitro and in vivo	Encapsulated curcumin significantly reduced in vivo tumor formation and significantly decreased the development of metastases by regulating apoptotic pathways. In vitro assay showed that both free and loaded curcumin decreased the survival and the ability of melanoma cells to generate colonies.	[90]
Polymeric nanoparticles (PLGA-PEG)	B16-F10 cells C57BL6 mouse model	Characterize and investigate the effects of curcumin and chrysin loaded into NPs on the expression levels of crucial genes with role in tumor progression and metastasis	The antimetastatic and antiproliferative effects of both polyphenols on melanoma cells in vivo and in vitro were improved when encapsulated in the PLGA-PEG polymeric NPs.	[91]
Liposomes	B16-F10 cells C57BL6J mouse model	Assess the inhibitory effect of curcumin loaded in modified liposomes in the tumor growth of a syngeneic mouse tumor model	This nanocomplex inhibited PI3K–Akt signaling pathways, causing the decrease in tumor growth.	[86]
	B16-BL6 cells	Investigate the in vitro skin permeation and in vivo antineoplastic effect of curcumin using different types of liposomes (soybean phospholipid liposomes (C-SPC-L), hydrogenated soybean phospholipids liposomes (C-HSPC-L) and egg yolk phospholipids liposomes (C-EPC-L))	C-SPC-L liposome showed to be the best liposomal formulation to inhibit the growth of B16-BL6 melanoma cells and is a promising transdermal carrier for curcumin in cancer treatment.	[92]
	B16-F10 cells C57BL6 mouse model	Evaluate the co-delivery of curcumin and anti-STAT3 siRNA using magnetic cationic liposomes	Liposomes were prepared with Fe_3_O_4_ and a mixture of N-didodecyl-glutamate chloride (TMAG) and dioleoyl phosphatidylethanolamine (DOPE) and loaded with curcumin. The positive charge on the liposome surface and the external magnetic field caused tumor progression inhibition.	[87]
Micelles	B16 and A375 cells C57 mouse model	Formulate curcumin-loaded MPEG-PLA (curcumin/MPEG-PLA) micelles in order to improve curcumin solubility and investigated its antitumor effect on melanoma in vitro and in vivo	Curcumin-loaded micelles induced higher percentage of apoptosis in both melanoma cell lines, while in tumor tissue, this nanocarrier inhibited neovascularization.	[88]
	B16-F10 cells	Formulate copolymeric micelles, methoxy-poly(ethylene glycol)-poly(D,L-lactide) (mPEG-PLA), to encapsulate curcumin, to improve its dispersibility and chemical stability and enhance its bioavailability	The Cur-mPEG-PLA nanosystem inhibited melanoma cell growth and was efficiently taken up by the cancer cells.	[93]
Solid lipid nanoparticle	B16-F10 cells	Use of chitosan to coat and stabilize solid lipid nanoparticles (SLNs) and then load the SLNs with curcumin	The modified SLNs with chitosan had significantly greater antitumor efficacy compared to free curcumin.	[94]
Au NPs	B16-F10 cells	Evaluate the combination of curcumin with NIR sensitive liposome gold nanoparticles (Au-Lipos Cur NPs) as an effective in situ adjuvant therapy for melanoma treatment	Due to the gold coating, the NPs absorbed NIR light (780 nm), and this light energy was converted to heat. The generated heat destabilized the liposomal core, enhancing the release of encapsulated curcumin. Cytotoxicity was also observed in the Au-Lipos Cur NPs-treated group after laser irradiation.	[95]
	B16-F10 cells C57/inbred mouse model	Synthesize, characterize and apply polyethylene glycol-curcumin-gold nanoparticles (PEG-Cur-Au NPs) for photothermal therapy	Induced tumors in the mice revealed a reduction in tumor volume upon photothermal therapy by PEG-Cur-Au NPs.	[96]
**Curcumin + Resveratrol**				
Solid lipid nanoparticle	B16-F10 and SK-MEL-28 cells	Develop a solid lipid nanoparticle for topical delivery to enhance the skin penetration and anticancer efficacy of curcumin and resveratrol	Curcumin and resveratrol solution was found to be more toxic than either drug solution alone.	[6]
**Quercetin**				
MSNs	JR8 cells	Evaluate the potential of aminopropyl-functionalized mesoporous silica nanoparticles (NH_2_-MSNs) as topical carrier system for quercetin delivery	MSNs showed absence of toxicity and good biocompatibility. The complex with NH_2_-MSNs was more effective than quercetin alone, causing inhibition of cell proliferation.	[21]
**Epigallocatechin-3-gallate**				
Polymeric nanoparticles (Chitosan)	Mel 928 cells Athymic (nu/nu) nude mouse model	Assess the antitumor efficacy of the formulated nano-EGCG in subcutaneously implanted tumor xenograft in athymic nude mice	Nano-EGCG showed better efficacy in comparison to free EGCG. Cells treated with nano-EGCG showed marked induction of apoptosis and cell cycle inhibition. Nano-EGCG also inhibited proliferation and induced apoptosis in tumors of the in vivo study.	[97]
Au NPs	B16-F10 cells C57BL6 mouse model	Investigate in vitro and in vivo the anticancer efficacy of EGCG-Au NPs on melanoma cells	Au NPs improved EGCG anticancer efficacy in melanoma cells, shown by increased cytotoxicity and apoptosis and inhibition of tumor growth.	[59]
**Apigenin**				
Polymeric nanoparticles (PLGA)	A375 cells	Evaluate the antiproliferative potential of apigenin loaded in PLGA nanoparticles (NAp)	NAp suppressed cell proliferation in a dose-dependent manner and induced apoptosis.	[66]
	B16-F10 cells C57BL6 mouse model	Develop a drug delivery system conjugating DMSA and apigenin-loaded in a PLGA nanosystem and evaluate its therapeutic potential to treat melanoma lung metastasis	Nanoformulation improved apigenin bioavailability with enhanced antitumor and antimetastatic efficacy.	[65]
**Genistein**				
Au NPs	HTB-140 cells	Develop a conjugate of gold nanoparticles and genistein (Au NPs-GE)	The treatment of the conjugate AuNPs-GE was more toxic than free genistein, suggesting that this nanocarrier could enhance the anticancer effect of genistein.	[89]
**Luteolin**				
Nanovesicles	B16-F1 cells	Prepare, characterize and optimize luteolin-loaded nanovesicles (LT-NVs) to be used as a potential delivery system in the treatment of melanoma	Optimized LT-NVs showed enhanced growth inhibitory effects in comparison to pure luteolin.	[5]
**Silymarin**				
Lipid nanocarrier	SK-MEL-2 cells	Formulate a nanostructured lipid carrier (NLC) system to increase the therapeutic value, anticancer action and reduced toxicity of silymarin	Silymarin-NLC proved to possess anticancer activity in a dose-dependent manner and the capacity to induce apoptosis.	[98]

## Data Availability

Not applicable.
